# An Investigation of Muscle Mechanical Properties in Acute Burns and Burn Types

**DOI:** 10.3390/jcm14072344

**Published:** 2025-03-29

**Authors:** Engin Ramazanoğlu, Serkan Usgu, Yavuz Yakut, Murat Ali Çınar, Kezban Bayramlar, Ali Güneş, Ahmet Erkiliç

**Affiliations:** 1 Physiotherapy and Rehabilitation Department, Faculty of Health Sciences, SANKO University, 27000 Gaziantep, Turkey; 2 Physiotherapy and Rehabilitation Department, Faculty of Health Science, Hasan Kalyoncu University, 27000 Gaziantep, Turkey; serkan.usgu@hku.edu.tr (S.U.); yavuz.yakut@hku.edu.tr (Y.Y.); muratali.cinar@hku.edu.tr (M.A.Ç.); kezban.bayramlar@hku.edu.tr (K.B.); 3 General Surgery, Burn Center, Gaziantep City Hospital, 27000 Gaziantep, Turkey; op.dr.ag1991@gmail.com (A.G.); aeres57@yahoo.com (A.E.)

**Keywords:** burn, biceps brachii, tone, stiffness, elasticity

## Abstract

**Objectives**: This study investigated the mechanical properties (tone, stiffness, and elasticity) of the biceps brachii (BB) muscle in the acute phase of different burn types. **Methods**: A total of 30 individuals (18–63 years) participated and were divided into three groups: flame, electrical, and scald burns. Myotonometric measurements assessed BB muscle tone, stiffness, and elasticity at admission (week 1) and at weeks 2 and 3. **Results**: When comparing the three time points, BB tone and stiffness significantly decreased by week 3 (*p* < 0.05), while elasticity remained unchanged (*p* > 0.05). Across burn types, BB tone, stiffness, and elasticity were similar at all three time points (*p* > 0.05). In the acute phase, BB tone and stiffness decreased by week 3, but elasticity showed no change. **Conclusions**: Different burn types exhibited similar BB mechanical properties over time. However, burn types may influence muscle tone and stiffness differently during acute recovery. Further studies with larger populations are needed to compare treatments across burn types and assess long-term mechanical property changes.

## 1. Introduction

The loss of resting skeletal muscle is one of the distinctive characteristics of the pathophysiological stress response observed in severe burns [1]. In severe burns (TBSA more than 20%), muscle atrophy and weakness make recovery difficult and are closely associated with morbidity and mortality [2,3]. Despite treatment approaches provided in the acute period in burn centers, muscle loss is observed and continues in the long term. After the burn, the body develops three different adaptive responses, the systemic inflammatory response, the stress response, and the hypermetabolic response, in which all of them are involved. An acute inflammatory response occurs with the increase in various pro-inflammatory cytokines as a result of damage to the skin, which is the main barrier to prevent infection [4]. There is limited information on hypercatabolism in muscles and how the above changes affect the mechanical properties (tone, stiffness, and elasticity) of the muscle. The use of objective evaluation methods may be important in explaining the loss of muscle, which is still controversial. It may also be valuable in terms of solving different prospective problems, physical fitness, and improving quality of life [3,4,5]. There is a need for objective, cost-effective, reliable, valid, and easy-to-use methods to evaluate the mechanical properties of muscles [6]. MyotonPRO (Müomeetria Ltd., Tallinn, Estonia) is a handheld device with these features. It provides a fast, non-invasive, cost-effective, and quantitative method for the evaluation of mechanical properties (tone, stiffness, and elasticity) [7]. The mechanical properties of muscles can be affected to varying degrees in different types of burns. In flame burns, a larger surface area is affected, which can lead to inflammation and fibrosis. Reductions in elasticity parameters may occur depending on the depth of the burn and the severity of fibrosis. It is not well known how scald burns, which are more superficial and localized, might affect muscle tone and stiffness. In electrical burns, deeper structures are affected, and it is suggested that reductions in elasticity parameters may occur in the long term due to scar tissue formation. No study investigating how muscle mechanical properties are affected after burns was found in the literature [8,9]. Based on the information in the literature, the hypothesis of this study was developed to determine how muscle mechanical properties change in acute burns and different burn types.

## 2. Patients and Methods

### 2.1. Study Design

This study was designed as a prospective observational study conducted at 25 Aralık State Hospital, Burn Center between [September/2018] and [March/2019]. A total of 52 individuals aged between 18 and 63 years who were admitted to the burn center were initially included in the study. The inclusion criteria for this study were determined as being right-hand dominant, having a burn in the right biceps brachii region, having a total body surface area burn of less than 50%, and being in the acute phase of burn injury (within days of injury). Individuals who underwent amputation after hospitalization, had a presence of chronic systemic diseases (e.g., diabetes mellitus, autoimmune diseases), were drug-addicted, and had a neuromuscular disorder that may affect muscle properties were excluded from the study.

**Figure 1:**
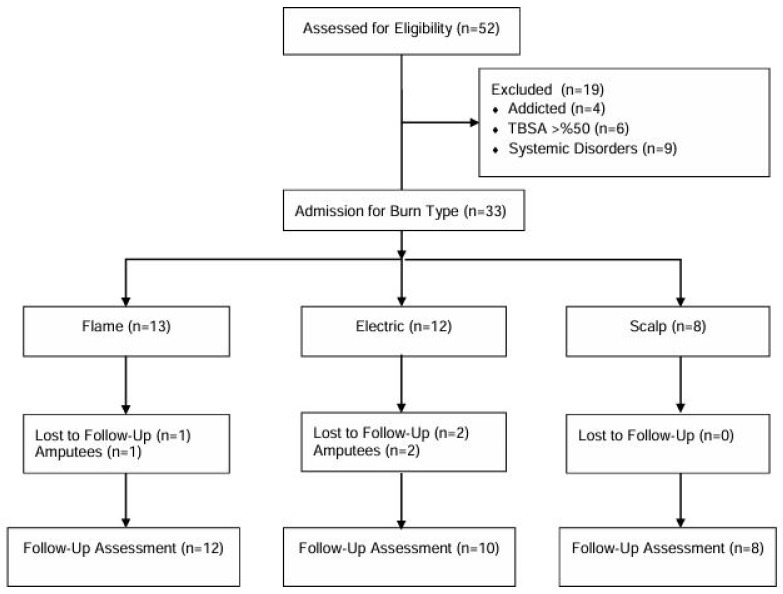
A flow chart of the study.

In our study, a total of 19 patients were excluded from the study, including 9 patients due to systemic diseases, 4 patients due to drug addiction, and 6 patients due to a burn percentage of more than 50%. In the follow-up of these excluded patients, after the initial assessment, 1 patient in the flame group and 2 patients in the electrical group underwent amputation. The study was completed with a total of 30 individuals (Figure 1).

After admission, the burn surface area and burn severity were determined by a specialist physician using the Rule of Nines. Data regarding burn localization, burn depth, and the pattern of occurrence were recorded. At the beginning of the study, demographic characteristics, including age, height, body weight, and body mass index (BMI), were collected. The individuals were categorized into three groups based on the burn type: flame burns (n = 10), electrical burns (n = 10), and scald burns (n = 10). All patients received standard acute wound care and medical treatment at the burn center. They were immobilized at similar levels during hospitalization and participated in a standard physiotherapy program, which included range of motion exercises, passive stretching, and mobilization exercises. The treatment protocol was identical for all patients but was adapted to suit the clinical condition of each individual. A dietitian at the burn unit ensured that all patients were provided with the same type of diet and nutrient composition to maintain consistency in metabolic effects across different burn types [10]. Patients were evaluated at three different time points: week 1 (baseline, upon admission), week 2 (weekly first dressing change), and week 3 (weekly first dressing change).

### 2.2. Evaluation of Creatine Kinase

Since creatine kinase (CK) is a marker of muscle breakdown and damage, its levels were measured to determine if there were differences among the three burn types. Venous blood samples were collected after a 12 h fasting period at each time point (weeks 1, 2, and 3). Blood collection was performed by a burn unit nurse with over 5 years of experience under the supervision of a specialist physician. The first blood sample was taken 24 h after the burn injury to standardize measurements [10]. All CK analyses were conducted in the biochemistry laboratory of the burn center using an automated enzymatic assay method (Siemens Dimension EXL, average for men: 52–336 U/L, average for women: 38–176 U/L). The samples were centrifuged at 3000 rpm for 10 min and stored at −80 °C before analysis.

### 2.3. Evaluation of the Mechanical Properties of the Biceps Brachii Muscle

**Figure 2:**
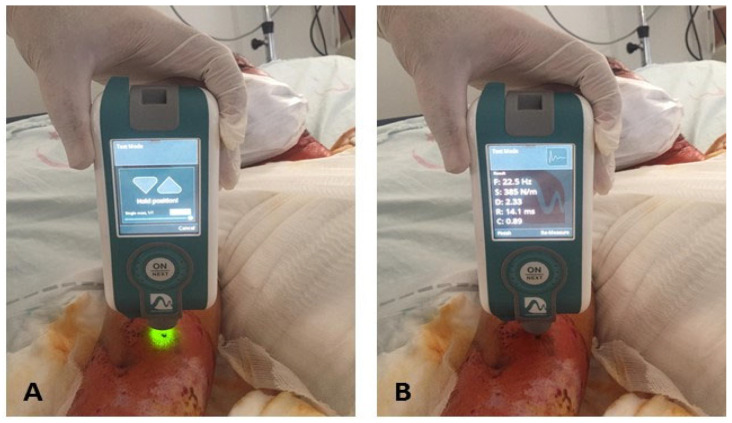
(**A**). Prior to the myotonometric measurement of the biceps brachii muscle, (**B**). The measurement result obtained from the reference point.

In the preliminary study we conducted on muscles on the burned and unburned sides, we observed that the mechanical properties were similar. Therefore, we evaluated the mechanical properties of the BB muscle, which we could frequently encounter in all three different types of burns. The mechanical properties were evaluated using the MyotonPRO device (Müomeetria Ltd., Tallinn, Estonia). MyotonPRO provides an objective and non-invasive digital measurement of soft tissues. Therefore, it can be used as a diagnostic and monitoring device in clinical practice [11]. The Myoton device can evaluate three different properties: Tone (Hz) characterizes the passive or resting tone of a muscle without any voluntary contraction [12]. Stiffness (N/m) refers to the resistance to an external force that distorts the contraction or original shape [12]. Elasticity (log) is the ability of the muscle to regain its original shape after the end of the contraction or external deformation force, and it is achieved by the logarithmic reduction in its natural oscillation [12]. The probe of the device (3 mm diameter) was placed vertically on the muscle with a constant load (0.18 N) to examine the subcutaneous tissues. After a short (15 ms) and low force (0.4 N) mechanical impulse that induced natural oscillations, the resting muscle tone, elasticity, and stiffness were obtained by using the accelerometer in the device [12]. The measurement was performed at ¾ of the distance between the lateral end of the acromion and the middle cubital fossa while the patient was lying in the supine position [11] (Figure 2). The arithmetic means of 3 consecutive measurements obtained from the reference point were used as data for each parameter. All measurements were performed in a temperature-controlled room (22–24 °C) at a fixed time (9:00–11:00 AM) to minimize circadian variation.

### 2.4. Statistical Analysis

The normality of distribution of continuous variables was tested by the Shapiro–Wilk test. One-way ANOVA (normal data), Kruskal–Wallis (non-normal data), and Dunn’s multiple comparison tests were used to compare numerical data across three groups. The Friedman test was performed for the comparison of non-normal data between 3 different time points. The chi-square test was applied to investigate the relationship between 2 categorical variables. Statistical analysis was performed with SPSS for Windows version 24.0, and *p*-value < 0.05 was accepted as statistically significant [13].

### 2.5. Ethical Consideration

This study was conducted in accordance with the principles outlined in the Declaration of Helsinki. All participants were fully informed about the study’s purpose, procedures, and potential risks. Written informed consent was obtained from all participants before data collection, and the study was carried out in compliance with The Strengthening the Reporting of Observational Studies in Epidemiology (STROBE) recommendations. Ethical approval dated 6 June 2018 and numbered 2018-05 was obtained from Hasan Kalyoncu University, Faculty of Health Science, Non-Invasive Research Ethics Committee to conduct this study.

## 3. Results

With regard to demographic characteristics, our groups were found to be similar in terms of sex distribution, age, height, body weight, body mass index, and burn percentage (*p* > 0.05) (Table 1).

The comparison of creatine kinase and mechanical properties in all burned patients is presented in detail in Table 2.

Table 3 and Table 4 present the intra- and intergroup comparison of muscle mechanical properties and creatine kinase in burn types.

## 4. Discussion

This study was conducted to investigate muscle mechanical properties (tone, stiffness, and elasticity) due to burns in the acute phase and whether these properties change in burn types. Among the muscle mechanical properties, tone and stiffness decreased in the acute phase. Burn types showed different mechanical properties within themselves during the recovery period.

### 4.1. In This Regard, We Consider That Our Study Is Valuable

Thirty burn patients aged between 18 and 63 years participated in our study. The total body burn percentages of the flame (30%), electrical (31.5%), and scald (20%) burns were similar. Although we obtained statistically similar TBSA values, we considered that the low value in scald burns was related to the formation mechanism.

### 4.2. Creatine Kinase (CK)

We considered that the fact that the CK value in the first week after the burn was higher in electrical burns, and then decreased in the third week and was similar to other types of burns, was especially due to the extensive damage to the muscular system caused by electrical burns. It was indicated that myocardial damage was rare and that the production of CK increased in addition to the stimulation of skeletal muscle in high-voltage electrical injuries [14]. In electrical burns, a level of CK above 400 U/L causes a longer hospital stay, skin graft, amputation, or mortality risk [15]. The values we found were close to the values of burn patients with amputation in another study [16].

### 4.3. Mechanical Properties (Tone, Stiffness, and Elasticity)

When we evaluated the electrical patients, we considered that the decrease in muscle tone and stiffness in the 3rd week might be due to the recovery of tissues as a result of the onset of recovery following systemic and intramuscular physiological changes that occurred in the acute phase. Stiffness depends on the muscle structure (length and cross-sectional area) and intramuscular contents [17]. The level of type 1 collagen protein is very low on the 7th day of the burn and starts to normalize on the 14th day. The excessive accumulation of intramuscular collagen is at its peak level 14 days after the burn and continues up to 21 days by decreasing, which reveals the fibrotic muscle phenotype that hinders regenerative capacity [18]. The ratio of collagen (type I/III) is significantly less and causes a decrease in tensile strength [19], which supports our results and may explain the decrease in BB stiffness, especially after the 2nd week. Tone can be divided into two groups, neural and non-neural forms. In burns, neural structures (the central and peripheral nervous system) can be affected, and acute and chronic diseases can be observed [20]. In particular, skin innervation, cell activation, and decreased signal transduction were demonstrated in burns [21]. In the acute phase, disruptions in the metabolic, vascular, endocrine, and microbiome systems are the mechanisms that affect the central nervous system, as well as the immune response [20]. The brain becomes the target of multiple complications, such as peripheral/central inflammation, nitric oxide imbalance, oxidative stress, and ultimately neuronal insulin resistance and insufficient glucose utilization in burns [22]. Mitochondrial dysfunction is actually at the center of these events [22]. The changes in the electroencephalogram are evidence of peripheral tissue damage and central nervous system disruption [23]. The spinal cord is the other target area. For the recovery of spinal cells, homeostasis causes motor neuron loss, synaptic degeneration, and muscle atrophy, especially as a result of microglia activation triggered by the inflammatory response [24]. It may be accompanied by decreased axon caliber in the peripheral nervous system and a slowing of conduction velocity [25]. Furthermore, tone (non-neural form) and stiffness may change with the physical activity level, contraction, exercise, or sports activities [26]. We considered that these neurodegenerative processes led to a decrease in muscle tone.

Interestingly, elasticity was the parameter that did not change among mechanical properties. There is actually a negative correlation between stiffness and elasticity. Elasticity increases as stiffness decreases. With recovery, scar tissue formations that will affect the elasticity of the muscle and the skin are expected to increase over time. The decrease in elasticity between weeks supports it, although this is not statistical. The presence of different methods and applications in the few studies that evaluate the elasticity in burns makes it difficult to compare our results.

When the groups divided according to burn types were compared, the mechanical properties of the BB were similar for weeks 1, 2, and 3. It was understood that tone, stiffness, and elasticity did not show different characteristics according to the burn type. However, they showed different mechanical characteristics in the recovery of burn types. We consider that this result is important for mechanical responses.

In particular, tissue damage is serious and progressive in electrical injuries, and vascular influence and thromboxane production lead to necrosis. The movement of electrons initiates an exothermic reaction, which is called electrolysis. It changes the pH and oxidation of the tissue. The denaturation of the cell membrane leads to the disruption of its semi-permeable structure, and the random flow of electrolytes is observed [27], which is typical in electrical burns and causes excessive effects on muscle tissue that is particularly resistant to current. The decrease in the stiffness and tone of the BB muscle immediately after the 2nd week may be associated with vascularization and the early recovery of membrane permeability. Furthermore, the occurrence of tetanic contractions in electrical burns may be another factor, which is especially related to the arterial and venous circulation. It is known that the mean blood flow and output to the muscle increase as a result of rhythmic contractions (tetanic contractions) [28]. These contractions may have caused a decrease in intramuscular edema in a short time by creating a pumping effect, and thus, the relaxation of the muscle tissue that is sensitive to mechanical compression.

The differentiation of tone, stiffness, and elasticity due to burn types during the recovery period indicates that the body is related to recovery strategies after the burn, and this may be important for different medical treatments or rehabilitation approaches.

According to the data we obtained from all groups, the severity of the muscle pathology, especially in major burns, seems to be associated with depth, as in flame burns, or the extent of tissue damage that cannot be determined from the outside, as in electrical burns, rather than the surface area of the burn. In other words, the destroyed tissue mass is more determinant. Therefore, the use of myotonometric measurements can be considered as an objective, fast, and non-invasive method for the diagnosis of burns, thus determining the recovery level of the patient and aiding in the planning of their treatments. They may be used especially in the diagnosis of some forensic cases of unknown burn type and in determining the size of tissue damage in electrical burns (in our study, tone and stiffness were high, although not statistically). Myoton can distinguish flash electrical burns, direct current injuries, and mixed injuries from each other in the first evaluation of the patient with a burn, which is supported by the fact that the CK values are parallel with the values obtained by Myoton. Furthermore, in direct current injuries, the entry, course, and exit areas of the current in the body can be detected quickly and accurately. In electrical burns, the burn is not visible superficially. It may not be possible to determine the exact extent of tissue damage in the clinic by investigating entry and exit wounds. Myotonometric measurements can provide important information about the progress of the electric current in the body, its severity, and how much of the body is affected by it. Thus, mortality and morbidity rates can be reduced by early interventions. Perhaps it can be determined whether the cases are exposed to high or low voltage current. Based on our observations, the changes in stiffness and tone were excessive in some of our patients who were exposed to very high electric currents. Based on these issues, it will be useful to investigate the mechanical properties in burns due to different current intensities. There is a need for studies with large populations in which the treatments applied in burn types are compared and the long-term outcomes of their mechanical properties are monitored.

If our patients had not been removed for surgical reasons, we could have had more information about the viscoelastic properties of the muscle. We consider that our study is original and important in terms of the quantitative evaluation of muscle tone, stiffness, and elasticity at the site of the burn for the first time.

## 5. Conclusions

This study investigated the mechanical properties (tone, stiffness, and elasticity) of the biceps brachii (BB) muscle in different types of burns during the acute phase. Our findings indicate that BB tone and stiffness significantly decreased by the third week, while elasticity remained unchanged. Interestingly, the mechanical properties did not show statistically significant differences between burn types (flame, electrical, and scald burns) at any of the three time points. However, burn types exhibited different recovery characteristics, especially in terms of muscle tone and stiffness. Among the burn types, electrical burns showed a more pronounced increase in creatine kinase (CK) levels in the first week, which later decreased and became similar to other burn types in the third week. This suggests that electrical burns cause extensive muscle damage initially, but tissue repair mechanisms become more active over time. Our results suggest that muscle mechanical properties are influenced not only by the burn type but also by systemic responses, including neural, metabolic, and vascular changes. The reduction in muscle stiffness and tone observed in the third week may be related to collagen remodeling, metabolic shifts, and neurophysiological adaptations. Despite the absence of statistically significant differences in mechanical properties between burn types, electrical burns appear to have unique characteristics related to muscle physiology, likely due to vascular compromise, altered nerve conduction, and tissue necrosis.

### 5.1. Limitations of the Study

This study has several limitations that should be addressed in future research: The study was conducted with a total of 30 patients, which may limit the generalizability of our findings. A larger sample size would enhance the reliability of the results. Our evaluation was limited to the first three weeks after the burn injury. Future studies should investigate the long-term effects of burns on muscle mechanical properties and functional recovery. Patients with more than 50% TBSA burns were excluded. The inclusion of these cases might provide additional insights into severe muscle damage and long-term functional outcomes. Although all patients received a standard physiotherapy protocol, individual variations in treatment response were not assessed. The effect of different rehabilitation strategies on muscle mechanical properties should be explored. Future studies should evaluate other biomarkers (e.g., inflammatory markers, oxidative stress parameters, and muscle enzymes) to provide a more comprehensive understanding of muscle pathophysiology in burn injuries.

### 5.2. Future Research Directions

Based on our findings, we propose the following directions for future research: Investigating the long-term progression of muscle tone, stiffness, and elasticity in burn patients beyond the acute phase (e.g., 6 months to 1 year post-injury). Examining the effectiveness of different physiotherapy and rehabilitation interventions on muscle recovery in burn patients. Exploring the correlation between CK levels, muscle mechanical properties, and functional outcomes in various burn types. Conducting comparative studies on muscle damage in high-voltage vs. low-voltage electrical burns to determine whether myotonometric measurements can assist in forensic analysis and early diagnosis. Using advanced imaging techniques (e.g., MRI, ultrasound elastography) to validate and complement myotonometric measurements in burn patients.

### 5.3. Clinical Implications

Our study highlights the potential use of myotonometric measurements as an objective, non-invasive, and rapid method for assessing muscle mechanical properties in burn patients. These measurements could be useful for the following areas: Monitoring recovery progress and guiding rehabilitation interventions. Differentiating between burn types and estimating tissue damage severity. Providing insights into the neuromuscular adaptations following burn injuries. In conclusion, this study provides a novel perspective on muscle mechanical properties in acute burns, emphasizing the need for larger, long-term studies to better understand the complex physiological changes occurring in burn patients.

## Figures and Tables

**Table 1:** The demographic characteristics of the participants.

	Flame (n = 12)	Electrical (n = 10)	Scald (n = 8)		
Sex	n (%)	n (%)	n (%)	χ^2^	*p*
Male	11 (91.7)	9 (90)	7 (87.5)	0.093	0.955
Female	1 (8.3)	1 (10)	1 (12.5)		
	Mean ± SD	Mean ± SD	Mean ± SD	F	*p*
Age (year)	30.83 ± 11.01	27.1 ± 6.08	34.63 ± 18.64	0.833	0.446
Height (m)	1.73 ± 0.09	1.72 ± 0.08	1.74 ± 0.08	0.150	0.861
Body Weight (kg)	77.5 ± 8.6	68.1 ± 13.08	76.5 ± 9.21	0.497	0.101
Body Mass Index (kg/m^2^)	26.12 ± 4.22	23.06 ± 4.86	25.16 ± 2.57	1.551	0.330
Burn Percentage (%)	30.17 ± 11.92	31.6 ± 12.12	20.13 ± 8.56	3.231	0.055

*p* < 0.05. Abbreviations: m: meter, kg: kilogram, SD: standard deviation, χ^2^: Chi-square test, F: One-way ANOVA test.

**Table 2:** Comparison of creatine kinase, BB tone, stiffness, and elasticity in all patients.

	Week 1 Mean (Min–Max)	Week 2 Mean (Min–Max)	Week 3 Mean (Min–Max)	χ^2^	*p*
Creatine Kinase (u/L)	302 [180–783] ^a,b^	153 [69–261]	104 [69–160]	16.75	0.001 *
Tone (Hz)	15.65 [14.4–16.5]	15.4 [14.1–16.7]	14.9 [14.3–15.4] ^b,c^	23.261	0.001 *
Stiffness (N/m)	267.5 [231–298]	257 [238–315]	238.5 [215–270] ^b,c^	20.267	0.001 *
Elasticity (log)	1.13 [0.93–1.33]	1.02 [0.82–1.15]	0.98 [0.92–1.18]	1.267	0.531

* *p* < 0.05. Abbreviations: BB: biceps brachii, Hz: hertz, N/m: Newton/meter, log: logarithmic decrement. The significant difference after the Kruskal–Wallis test results and post-hoc pairwise (Dunn correction) comparisons output of the Friedman test. ^a^ *p* < 0.05 week 1 vs. week 2. ^b^ *p* < 0.05 week 1 vs. week 3. ^c^ *p* < 0.05 week 2 vs. week 3.

**Table 3:** Intra- and intergroup comparison of creatine kinase in burn types.

	Flame (n = 12)	Electrical (n = 10)	Scald (n = 8)	Intergroup Comparison	
	Median [25–75%]	Median [25–75%]	Median [25–75%]	H	*p*
Creatine Kinase (u/L)					
Week 1	228.5 [119–393]	2216 [783–2719] ^a,c^	226 [92–306]	15.197	0.001 *
Week 2	112.5 [61.5–167]	258.5 [83–670]	123 [61.5–208.5]	3.508	0.173
Week 3	109.5 [83.5–325]	103.5 [45–130]	97.5 [71–159.5]	1.380	0.502
Intragroup Comparison	χ^2^ = 2.17, *p* = 0.338	χ^2^ = 16.20, *p* = 0.001 *	χ^2^ = 7.03, *p* = 0.030 *		

* Significant at the 0.05 level, H: The Kruskal–Wallis test for intergroup comparisons, χ^2^: The Friedman test for intragroup comparisons. The significant difference after the Kruskal–Wallis test results and post-hoc pairwise (Dunn correction) comparisons output of the Kruskal–Wallis test. ^a^ *p* < 0.05 flame vs. electrical. ^c^ *p* < 0.05 electrical vs. scald.

**Table 4:** Intra- and intergroup comparison of muscle mechanical properties in burn types.

	Flame (n = 12)	Electrical (n = 10)	Scald (n = 8)	Intergroup Comparison	
	Median [25–75%]	Median [25–75%]	Median [25–75%]	H	*p*
BB Tone (Hz)					
Week 1	15.65 [14.15–16.2] ^a,b^	16.2 [14.6–17.3]	15.05 [14.15–16.45]	1.120	0.571
Week 2	15.15 [13.45–16.45]	16.2 [15.3–18.3]	15.25 [14.15–15.65]	3.206	0.201
Week 3	14.65 [14–15.3]	15 [14.4–17] ^b,c^	14.7 [14.15–15.05]	1.040	0.595
Intragroup Comparison	χ^2^ = 8.67, *p* = 0.013 *	χ^2^ = 12.20, *p* = 0.002 *	χ^2^ = 5.25, *p* = 0.072		
BB Stiffness (N/m)					
Week 1	277 [210.5–318]	276.5 [237–285]	235 [217.5–281]	0.759	0.684
Week 2	265 [231.5–313.5]	257.5 [243–330]	250.5 [220–276]	0.643	0.725
Week 3	234.5 [198.5–282] ^b,c^	241 [219–270] ^b,c^	236.5 [220–264]	0.461	0.794
Intragroup Comparison	χ^2^ = 13.17, *p* = 0.001 *	χ^2^ = 9.60, *p* = 0.008 *	χ^2^ = 0.75, *p* = 0.687		
BB Elasticity (log)					
Week 1	1.18 [0.98–1.37]	1.15 [0.79–1.22]	1.07 [0.98–1.46]	0.977	0.614
Week 2	1.1 [0.84–1.32]	0.92 [0.81–1.04]	1.05 [0.88–1.22]	2.225	0.329
Week 3	1.06 [0.93–1.24]	0.98 [0.9–1.05]	0.99 [0.96–1.11]	0.274	0.872
Intragroup Comparison	χ^2^ = 1.17, *p* = 0.558	χ^2^ = 0.80, *p* = 0.670	χ^2^ = 0.25, *p* = 0.882		

* *p* < 0.05. Abbreviations: BB: biceps brachii, Hz: hertz, N/m: Newton/meter, log: logarithmic decrement. The significant difference after the Kruskal–Wallis test results and post-hoc pairwise (Dunn correction) comparisons output of the Friedman test. Intragroup comparison; ^a^ *p* < 0.05 week 1 vs. week 2. ^b^ *p* < 0.05 week 1 vs. week 3. ^c^ *p* < 0.05 week 2 vs. week 3.

## Data Availability

The data presented in this study are available on request from the corresponding author. The data are not publicly available due to ethical and legal restrictions, as they contain potentially identifying and sensitive patient information.
